# CAR-NK cell immunotherapy: a comprehensive review of engineering platforms, clinical applications in hematological and solid tumors

**DOI:** 10.1007/s00432-026-06635-1

**Published:** 2026-10-23

**Authors:** Salma Mohamed Awadallah, NourEldin Elmesalamy, Ahmed Reda Bahr, Basant Atef, Eeman Afroz, Hank C. Hill

**Affiliations:** 1 Faculty of Medicine, Benha National University, Obour, Egypt; 2 Faculty of Medicine, Alexandria University, Alexandria, Egypt; 3 Faculty of Medicine, Tanta University, Tanta, Egypt; 4 Allama Iqbal Medical College, Lahore, Pakistan; 5 Assistant Professor of Surgery, Director of Breast Surgical Services, Texas Tech University Health Sciences Center, El Paso, USA

**Keywords:** Natural killer cells, Chimeric antigen receptor, CAR-NK, Immunotherapy, Cellular therapy, Cancer

## Abstract

Chimeric antigen receptor–natural killer (CAR-NK) cells combine innate cytotoxicity with antigen-directed killing and promise as an immunotherapy with lower cytokine toxicity than CAR-T approaches. However, clinical translation is limited by challenges in transduction, persistence, tumor infiltration, and the immunosuppressive tumor microenvironment (TME). We performed a comprehensive literature review of recent preclinical and early-phase clinical studies, engineering strategies (CAR design, transduction technologies, gene-editing), manufacturing and expansion protocols, and trial outcomes in this review. We noted a reference to the advantages of CAR-NK, like multimodal killing (CAR-dependent and innate pathways), potential for allogeneic “off-the-shelf” use, and favorable early safety profiles. However, NK-specific obstacles persist: lower viral transduction efficiencies, short in vivo persistence, poor infiltration into solid tumors, and TME-mediated suppression (TGF-β, adenosine, metabolic stress). Engineering countermeasures such as IL-15 expression, checkpoint receptor knockout, chemokine receptor co-expression (e.g., CXCR1/4), DAP10/DAP12-based signaling domains, and non-viral delivery (transposons/CRISPR) have shown preclinical promise. CAR-NK therapy has developed from concept to a clinically actionable treatment line for hematologic malignancies and shows emerging potential in solid tumors when combined with TME-modulating strategies. This review provides robust evidence about CAR-NK therapies in hematological and solid tumors, with a great emphasis on the challenges they encounter, strategies, and innovative approaches to overcome these challenges, and forward-looking perspectives to improve future therapeutic CAR-NK designs and clinical outcomes.

## Introduction

Natural Killer (NK) Cells, specialized cells within the innate lymphoid cell (ILC) family, make significant contributions to the initial stages of host defense, including cytotoxic effects against infected, foreign, and tumor cells (Zhong and Liu 2024a). NK cells demonstrate a potent cancer therapy and pathogen removal without the required prior recognition or sensitization of the peptide antigens, which makes them more preferred than T lymphocyte-based cytotherapy (L. Zhang et al. 2022a, b). Firstly, in an in vitro study on leukemia, NK cells were identified as a distinct lymphocyte subset capable of spontaneous, MHC-independent cytotoxicity against tumors, a discovery made by Kiessling et al., representing the conceptual foundation for their later exploitation as a cellular immunotherapy platform (Kiessling et al. 1975). Then, NK cells were discovered in lymphoid tumors with their reactivity and cytotoxicity against several different antigens (Herberman et al. 1975).

NK cells can differentiate between healthy tissues and pathological cells. This mechanism results from a combination of both stimulatory and inhibitory surface receptors. NK cell identification of cancerous cells is controlled by the communication of particular ligands on the cancerous cells (Karamivandishi et al. 2025). To contribute to the overall immune surveillance, NK cells infiltrate almost all body organs. For example, the liver not only utilizes its resident NK cells but also recruits external NK cells to avoid any possible chronic inflammation. CXCR6, a specialized chemokine receptor, plays an important role in NK cell retention in the liver. Similar mechanisms to guide NK cells are also present in other organs (Stegmann et al. 2016). Incidentally, most tumor cells are capable of evading T cell immunity utilizing downregulation of MHC-I molecules; however, this makes them susceptible to NK cell surveillance. NK cells recognize alternative stress-induced ligands such as MICA/MICB and UL16-binding proteins so that they can target such immune-evasive cancers efficiently (Tufail et al. 2025; Tyshchuk et al. 2025).

NK cell development is guided in bone marrow under a process called “education”. which induces through selective expansion of NK cells capable of responding to major histocompatibility complex class I (MHC-I) interactions through immunoreceptor tyrosine inhibitory motifs (ITIMs) (Boudreau et al. 2016; Dhatchinamoorthy et al. 2021). Phenotypically, NK cells are, in general, subclassified into two functional subsets: CD56 bright NK cells, which are cytokine-producers mainly, and CD56 dim NK cells, which are cytotoxic and are also CD16 and killer-cell immunoglobulin-like receptor (KIR)-expressing (Flemming 2024).

NK cell-mediated cytotoxicity is primarily executed by perforin and granzyme release, as well as by death receptor pathways like Fas ligand (FasL) and tumor necrosis factor-related apoptosis-inducing ligand (TRAIL). NK cells also play a critical immunomodulatory role through cytokine supply, like interferon-gamma (IFN-γ) and tumor necrosis factor-alpha (TNF-α), which participate in anti-tumor immunity via the recruitment of dendritic cells (DC) and bridging innate and adaptive immune responses (Chen et al. 2024; Screpanti et al. 2005). Its functional activity is controlled through a set of inhibitory receptors (e.g., KIRs, CD94/NKG2A) and activating receptors (e.g., NKG2D, natural cytotoxicity receptors), enabling the targeted differentiation between normal and malignant cells. Crucially, NK cells are also capable of antibody-dependent cellular cytotoxicity (ADCC) via CD16, further adding to their therapeutic potential (Greppi et al. 2024; Kaulfuss et al. 2023).

In hematological malignancies such as acute myeloid leukemia (AML), NK cell cytotoxicity was strongest, especially when KIR mismatches enhance target recognition. Nevertheless, there are limitations to the application of NK cells to solid tumors, maybe due to their inability to infiltrate the tumor microenvironment (TME) and sensitivity to its immunosuppression. Despite these limitations, higher intertumoral numbers of NK cells have been associated with a favorable prognosis in many cancer types, emphasizing their potential as a prognostic and therapeutic agent (Lim et al. 2025).

This review comprehensively covers CAR-NK therapies from the mechanistic strategies of cancer evasion and underscores the emergence of CAR-NK therapy, from preclinical to clinical applications in hematological and solid tumors. Additionally, the review extensively investigates the superiority of CAR-NK over CAR-T, with a great emphasis on the challenges they encounter, strategies, and innovative approaches to overcome these challenges, and forward-looking perspectives to improve future therapeutic CAR-NK designs and clinical outcomes.

## Mechanistic strategies of cancer evasion from NK cells

Despite the inherent cytotoxic capacity of NK cells, tumor cells have evolved multifaceted strategies to evade NK cell-mediated immune recognition. One of the strategies involves the release of suppressive cytokines such as interleukin-10 (IL-10) and transforming growth factor-beta (TGF-β) that suppress NK cell proliferation, activation, and cytotoxic activity (Coënon et al. 2024; Kuznetsova et al. 2025). Tumors can also downregulate tumor-associated antigens (TAAs) or MHC class I molecules to evade immune detection, paradoxically generating NK activity while still being capable of inhibiting overall immune capability (Jhunjhunwala et al. 2021; S. Kong et al. 2025a, b). Within the tumor microenvironment (TME), an interplay of cellular and molecular elements exists that further suppresses the activity of NK cells. Tregs (Regulatory T cells), MDSCs (Myeloid-derived suppressor cells), CAFs (Cancer-associated fibroblasts), and tDCs (Tolerogenic dendritic cells) are some of the elements that contribute to the creation of such an immunosuppressive environment by secreting inhibitory cytokines and upregulating immune checkpoint expression. These, in combination, inhibit NK cell receptor expression and cytotoxicity, significantly downregulating activating receptors NKG2D, NKp30, and CD16, but upregulating inhibitory receptors NKG2A, tipping the balance of signals toward immune tolerance (Zhong and Liu 2024a).

In addition, MDSCs play a crucial role in modulating NK cell functions by inhibiting Fc receptor-mediated ADCC, cytokine production, and signal transduction. NK cells at advanced-stage tumors express lower levels of main activating receptors, and sustained exposure to immunosuppressive cytokines and metabolic stress (e.g., TGF-β and IL-10) further weakens their function (He et al. 2025; Tumino et al. 2021; Wang et al. 2025). Chemokine signaling is also implicated in immune suppression, as CCR4 expression on the cells of non-small cell lung carcinoma (NSCLC) allows for recruitment of Tregs by CCL17 and CCL22 and inhibits NK cell access to the tumor site (He et al. 2026; Lian et al. 2026).

At the cellular level, tumor-infiltrating NK cells also have phenotypic and metabolic defects, such as mitochondrial fragmentation, that render them non-viable and non-tumor-killing. This has been blamed on the hypoxic TME, which invokes the mTOR–Drp1 pathway, resulting in mitochondrial dysfunction. Inhibiting this pathway has been proven to restore NK cell mitochondrial integrity and cytotoxicity (Ghosh et al. 2025; Miao et al. 2024). Other mechanisms of immune evasion involve tumor-secreted enzymes such as indoleamine 2,3-dioxygenase (IDO1), which breaks tryptophan into kynurenine, to support the proliferation of Treg and suppress NK cells. The immune evasion is also achieved by tumor cells by hijacking Toll-like receptor 4 (TLR4) signaling to generate factors IL-6, IL-12, iNOS, and B7 family ligands, increasing cytotoxic lymphocyte resistance and establishing feedback mechanisms that promote immune evasion (Sun et al. 2025; Zhang et al. 2024). Lastly, tumors such as glioblastomas secrete soluble lactate dehydrogenase 5 (LDH5), which induces expression of NKG2D ligands on normal myeloid cells. This imposter blockade overburdens NKG2D receptors on NK cells and prevents effective targeting of tumor cells expressing NKG2D ligands (Guyon et al. 2022; Mohan et al. 2021).

Collectively, these multifaceted evasion mechanisms highlight the complexity of tumor–NK cell interactions and reaffirm the need for genetically engineered strategies—such as CAR-NK cells—to overcome such barriers to restore anti-tumor immunity. Although NK cells can detect and eliminate most cancer cells, some cancer cells have advanced their mechanisms to avoid detection by NK cells or block NK cell activities. For instance, cancer cells secrete immunosuppressive molecules (eg, interleukin-10 [IL-10] and transforming growth factor-beta [TGF-β]), which suppress NK cell function (Portale and Di Mitri 2023).

## CAR-NK cells: from biological properties to therapeutic mechanisms

### Biological basis and engineering of CAR-NK cells

CAR-NK cells are genetically modified to recognize specific tumor markers and are valued for their safety profile, especially in treating blood cancers. Noting that these therapies face distinct developmental and practical challenges. The production of CAR-NK cells is complex, and the field is still in early stages; ultimately, the choice of therapy must be tailored to the individual, considering the cancer's type and stage, as well as the patient's overall health. The continued development of these treatments is steadily expanding the arsenal of options available for fighting cancer (Ren et al. 2026; Xu et al. 2026).

CAR-T and CAR-NK cells can both be genetically modified to enhance their ability to identify target cells to be killed. Research has experimented with several delivery methods, such as transposon systems, lentiviral or retroviral-based transduction, and mRNA electroporation, demonstrating the versatility of CAR-NK technology. CD28, CD3ζ, 4-1BB, NKG2D, 2B4, and DNAM1 are all TCR co-stimulatory molecules that include the signaling domains of CAR-NK cells, which are similar to those of CAR-T cells (Honikel and Olejniczak 2022; Jayaraman et al. 2020; C. Liu et al. 2026b, a). Over successive generations, CAR designs have evolved as follows: The First generation has only CD3ζ, which is limited in persistence and signaling. On one hand, the Second generation was the same as the first generation, but with the addition of one co-stimulatory domain (CD28, 4-1BB). On the other hand, the third generation has two co-stimulatory domains that enhance activation and memory potential. Then the Fourth generation (TRUCKs) has been engineered for cytokine secretion (IL-2, IL-12, IL-15, IL-18) to modulate the tumor microenvironment. Lastly, the Fifth generation incorporated truncated cytokine receptor domains to activate JAK/STAT pathways in an antigen-dependent manner (Alsaieedi and Zaher 2025; Minguet et al. 2025).

### Therapeutic mechanisms and strategies to improve CAR-NK cells

**Fig. 1:**
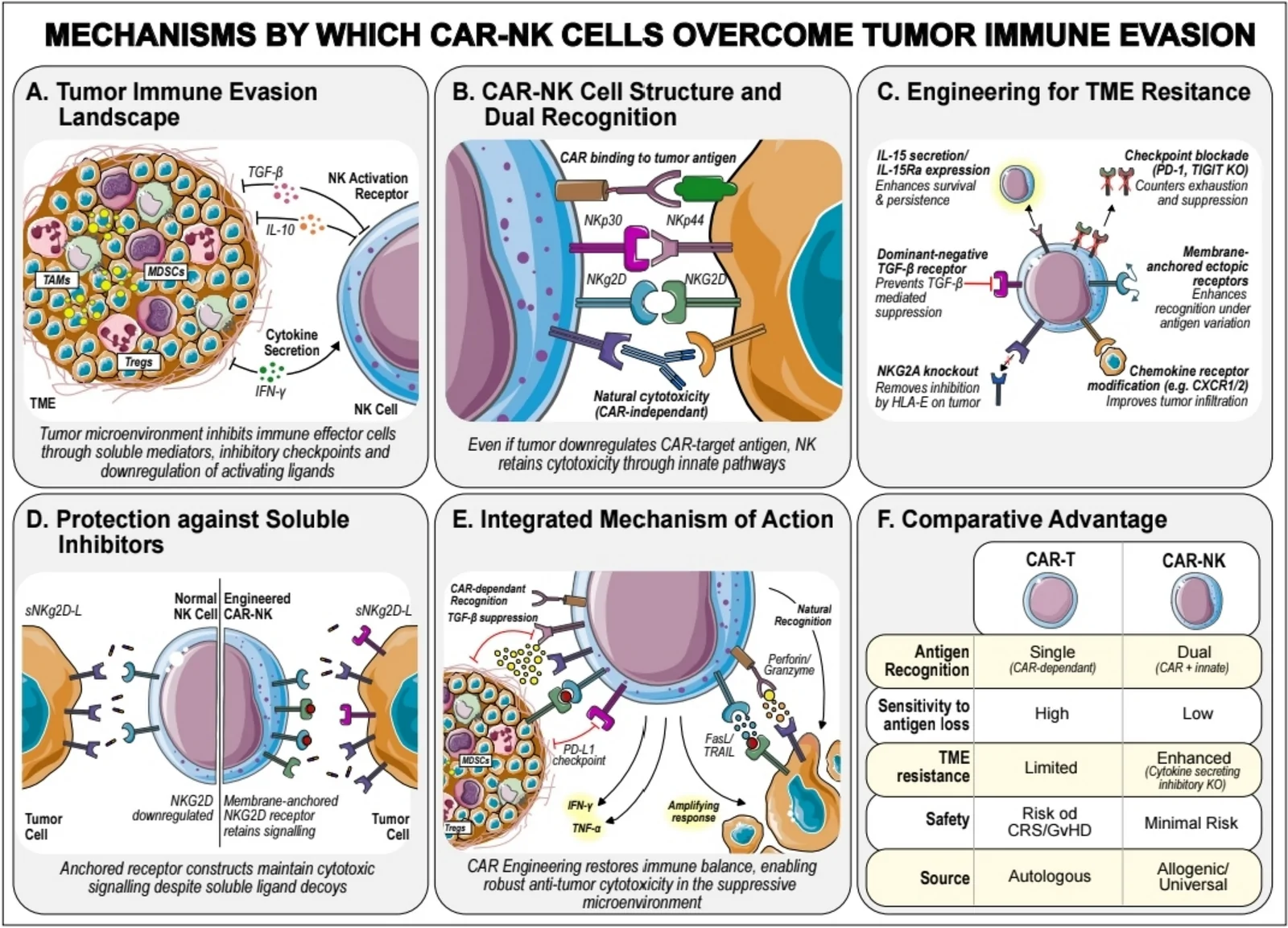
Illustration of the mechanisms by which CAR-NK overcome tumor immune evasion. Icons have been adapted and recreated from Servier Medical Art (https://smart.servier.com/), licensed under CC BY 4.0 (https://creativecommons.org/licenses/by/4.0/). This figure illustrates how chimeric antigen receptor-natural killer (CAR-NK) cells overcome tumor immune evasion within the tumor microenvironment (TME). Tumor-mediated suppression through transforming growth factor-beta (TGF-β), interleukin-10 (IL-10), inhibitory receptors, and metabolic stress impairs natural killer (NK) cell activity. CAR-NK cells counteract these mechanisms through major histocompatibility complex-independent targeting, innate activating receptors, and engineered modifications, including interleukin-15 (IL-15) expression and checkpoint receptor blockade. Tumor elimination occurs through perforin/granzyme release, Fas ligand (FasL), tumor necrosis factor-related apoptosis-inducing ligand (TRAIL), and antibody-dependent cellular cytotoxicity (ADCC). Abbreviations: CAR-NK, chimeric antigen receptor-natural killer; TME, tumor microenvironment; IL-15, interleukin-15; ADCC, antibody-dependent cellular cytotoxicity

Their inherent biology makes NK cells uniquely suited for this role. Their activation state is determined by a balance of activating receptors (e.g., NKG2D, NKp30, NKp44) that bind to stress-induced ligands on target cells, and inhibitory receptors (e.g., KIRs) that recognize healthy HLA-I molecules, allowing them to identify and eliminate abnormal cells early (F. Zhang et al. 2025a, b). A significant advantage of CAR-NK cells over CAR-T cells is that they possess both CAR-dependent and CAR-independent cytotoxic pathways, including natural cytotoxicity receptors and antibody-dependent cellular cytotoxicity (ADCC). This dual capacity allows them to target tumors even if the specific CAR antigen is downregulated, potentially making them more efficacious (Hassan et al. 2024). Preserving this natural function is a key benefit that helps prevent tumor immune evasion (Fig. 1).

**Fig. 2:**
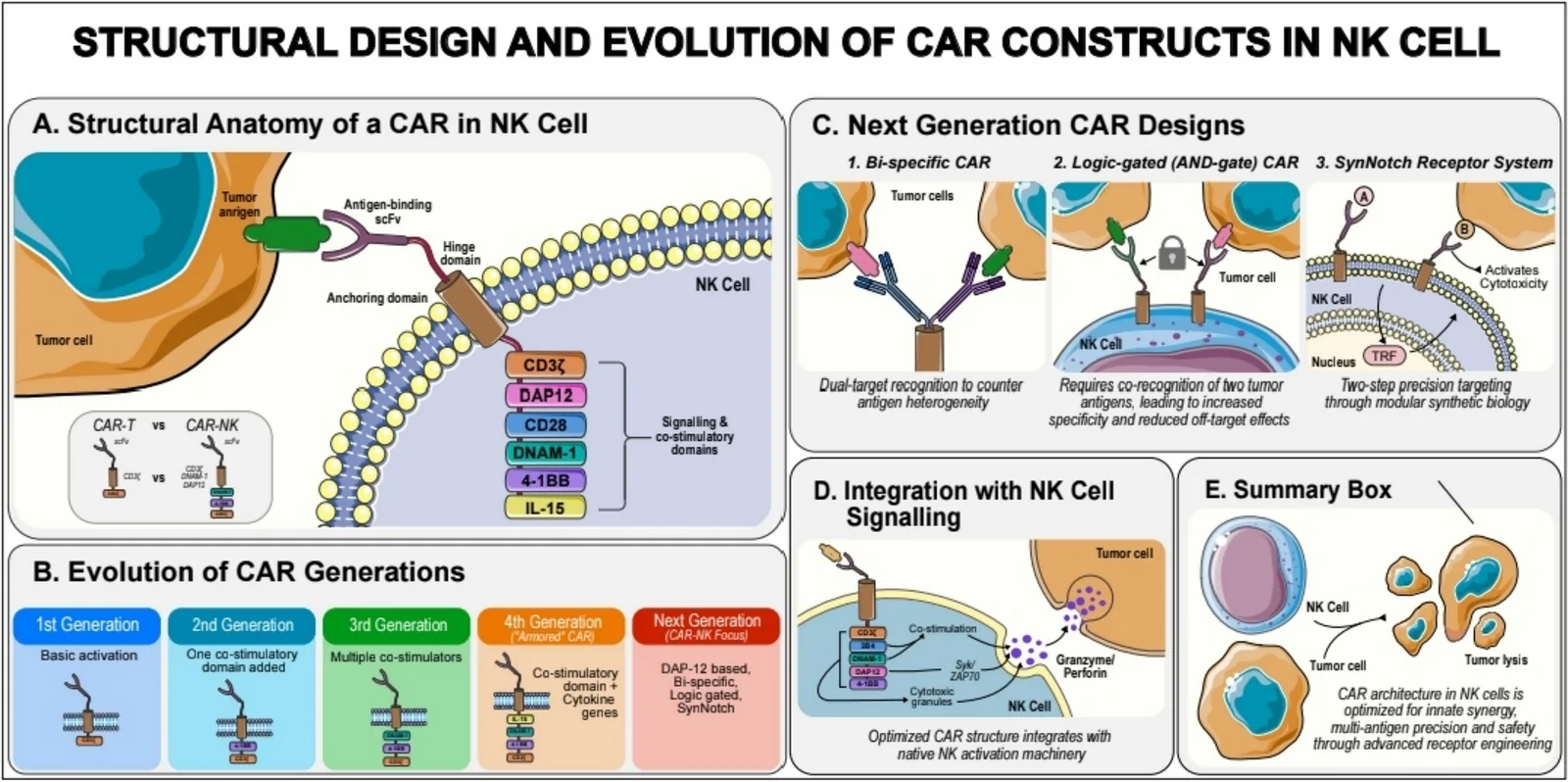
Illustration for the structural design and evolution of CAR in NK cell. Icons have been adapted and recreated from Servier Medical Art (https://smart.servier.com/), licensed under CC BY 4.0 (https://creativecommons.org/licenses/by/4.0/). This figure illustrates the structural components and generational evolution of chimeric antigen receptor-natural killer (CAR-NK) cells. CAR constructs consist of an extracellular antigen-binding domain, hinge region, transmembrane domain, and intracellular signaling motifs that activate NK-cell cytotoxicity. Successive CAR generations incorporate co-stimulatory domains, cytokine-secreting modules, and Janus kinase/signal transducer and activator of transcription (JAK/STAT)-activating elements to enhance persistence and antitumor efficacy. NK-specific signaling adaptors, including DNAX-activating protein 10 (DAP10), DNAX-activating protein 12 (DAP12), and 2B4 (CD244), further optimize NK-cell activation. Abbreviations: CAR, chimeric antigen receptor; NK, natural killer; JAK/STAT, Janus kinase/signal transducer and activator of transcription

While NK cells with the DAP12 construct displayed the greatest cytolytic activity, chimeric receptors in NK cells using the NKG2D ectodomain, when combined with either of the DAP12 or CD3ζ signaling domains, were both functioning well (Ng et al. 2021). More data about the NKG2D proved that it can evade the inhibitory receptor-induced signal to activate NK cells. For CAR-NK to be successful in fighting solid tumors, the NKG2D pathway needs to be capable of influencing tumor initiation and development (Wang et al. 2024). (Fig. 2).

In addition to direct cytotoxicity against certain antigens of tumor cells, CAR-NK cells can also diminish immunosuppressive cells from the TME. Targeting M2-like tumor-associated macrophages (TAMs) and myeloid-derived suppressor cells (MDSCs) are examples of some therapeutic approaches. Another suggestion to further modify cancer immunotherapy tactics is the combination of CAR-NK cells with T cell-based therapies (T. Li et al. 2024a; Wang et al. 2024; Zhong and Liu 2024a). In favor of patients with hematologic malignancies, autologous tumor cells that were previously resistant to the cytotoxic effects of NK cells without the CAR can be effectively eliminated by emerging approaches. It is worth mentioning that it is now possible to further personalize NK cells by introducing CARs and inactivating inhibitory genes like TIGIT and KLRC1(Chen et al. 2025; W. Liu et al. 2026b, a; Shang et al. 2024).

Interestingly, human leukocyte antigen (HLA) matching and prior exposure to tumor-associated antigens (TAAs) are not vital to enable the use of CAR-NK cells. Considering these redirected effector cells can be utilized to create "off-the-shelf" anti-cancer immunotherapeutic remedies(Damian 2026; Oseni et al. 2025).

To improve the CAR-NK cells regarding their persistence, the following strategies were tried. For example, the CAR-NK cells were made to create cytokines by genetically altering them, especially IL-15, which has proven to be of great assistance in all regards, especially in the persistence regard. IL-2 and IL-15 are the two main cytokines utilized to improve the survival and durability of NK cell treatments; a quick comparison between them would declare IL-15 the better option for the mission, due to its better toxicity profile and lack of support for Treg proliferation. To improve in vivo persistence, modern CAR-NK methods of genetic engineering have begun introducing genes that permit the ectopic expression of cytokines such as IL-15 (Gao et al. 2026; Vahidi et al. 2024). A second tactic was tried in which the apoptotic signals of the tumor cells were targeted, or pro-apoptotic genes in CAR-NK cells were eliminated using gene-editing tools. As a result, they may become more resistant to the TME's apoptotic signals. A third method involved altering the CAR construct by incorporating co-stimulatory domains that, when engaged with their target antigen, can produce strong survival signals (Zhong and Liu 2024b).

On the practical front, Zhu et al. indicated that prior activation with some cytokines could result in a memory-like state in CAR-NK cells, leading to higher persistence and antitumor activity in vivo. In comparison to regular NK cells, these memory-like CAR-NK cells displayed a gene expression profile linked to a prolonged lifespan and solid activity (Y. Huang et al. 2025a, b; Sanchez-Correa et al. 2019).

### Comparison between CAR-NK and CAR-T cell therapies

CAR-T therapy has achieved landmark successes in hematologic malignancies such as B-cell acute lymphoblastic leukemia (B-ALL), diffuse large B-cell lymphoma (DLBCL), and multiple myeloma, with its several FDA-approved agents (e.g., tisagenlecleucel, axicabtagene ciloleucel) employing second-generation constructs, however that, its limitations include manufacturing complexity, delayed treatment timelines, high cost, risk of severe toxicities (cytokine release syndrome, neurotoxicity), and tumor escape through antigen loss or immune suppression (X. Zhang et al. 2022a, b; Zugasti et al. 2025; Hernández-Idarraga et al. 2026). Despite promising findings of CAR-T cell therapy with relapsed or refractory blood cancer, it is still being challenged by significant obstacles that do not allow it to achieve its full potential: CAR-T cell generation is a very personal procedure: T cells of the individual patient have to be harvested, genetically engineered, cultured, and reintroduced. This personalized production line is convoluted, time-intensive, and expensive, sometimes more than 400,000 dollars per treatment in the United States (Patel et al. 2025). In the case of most patients, particularly those who have previously experienced several cycles of chemotherapy, healthy T cells are hard to get, resulting in time delays that may deteriorate the condition (Das et al. 2019; Jo et al. 2023). Meanwhile, the CAR-NK cells also have biological versatility. They can eliminate tumor cells that have down-regulated the expression of the MHC class I- a frequently used method to escape T-cell-mediated cell death- and are capable of effecting antibody-dependent cellular cytotoxicity (ADCC) through the CD16 receptor (W. Wang et al. 2024a; Zhong and Liu 2024a, b, ) **[**Table 1**].**

**Table 1:** CAR-T cells versus CAR-NK Cells: a comparative analysis of cellular immunotherapies

Feature	CAR-T Cells	CAR-NK Cells
Cell source	Autologous T cells (patient-derived) or allogeneic donors	Peripheral blood, cord blood, iPSC-derived, NK-92 cell line
Mechanism of action	CAR-mediated antigen recognition + TCR signaling	CAR-mediated antigen recognition + innate NK receptor-mediated killing
Generations	1st to 5th gen CARs; evolving towards armored and logic-gated designs	Adaptations from CAR-T designs with NK-specific signaling domains (DAP10, DAP12, 2B4)
Manufacturing	Patient-specific, time-intensive (2–4 weeks)	“Off-the-shelf” possible with allogeneic or cell line-derived NK cells
Toxicity profile	Higher incidence of CRS, neurotoxicity, on-target/off-tumor effects	Lower CRS and neurotoxicity risk, minimal GVHD
Persistence	Long-term engraftment possible, especially with memory T cells	Shorter lifespan in vivo, requires optimization for persistence
Tumor targets	Primarily hematologic malignancies; limited solid tumor efficacy	Active research in hematologic and solid tumors
FDA-approved products	Multiple (e.g., tisa-cel, axi-cel, liso-cel, ide-cel)	None yet (ongoing clinical trials)
Key advantages	Proven clinical efficacy, durable responses in blood cancers	Reduced toxicity, scalable production, dual killing mechanisms
Key limitations	High cost, manufacturing delays, toxicity, antigen escape	Short persistence, antigen expression requirement, mostly preclinical

Another challenge to CAR-T therapy is safety: the use of CAR-T therapy may cause severe immune reactions, such as cytokine release syndrome (CRS), neurotoxicity, tumor lysis syndrome, and graft-versus-host disease (Li et al. 2024a, b). For more explanation: CRS is caused by excessive immune response, which results in excessive production of inflammatory molecules, including IL-6, IFN-γ, and TNF-α, which may cause fever, low blood pressure, and even multi-organ failure, the causes of neurotoxicity, which occur in as many as 50 percent of patients receiving treatments, are believed to be caused by the inflammation due to cytokines that impair the blood–brain barrier. Although tocilizumab and corticosteroids are useful in alleviating such complications, they occasionally decrease the anti-cancer efficacy of the therapy. Collectively, CAR-T cells are coupled with limited success in relapse cases, highlighting the great need to have safer, faster, and cheaper immune cell therapies. In contrast to CAR-T cells, CAR-NK cells have a lower risk of causing severe CRS or neurotoxicity, and they do not cause graft-versus-host disease. Their cytokine profile is also less toxic—abundant in IFN-7 and GM-CSF but low in IL-6, which explains their decreased effects of inflammation (L. Zhang et al. 2022a, b; X. Zhang et al. 2022a, b; Luo et al. 2026).

### CAR-NK cell therapy in hematological malignancies: mechanisms of action and clinical outcomes

#### Leukemia

Leukemic cells have the ability to escape NK cell attacks. This may be attributed to a defect in NK cells themselves or to the immunosuppressive privilege of the tumor microenvironment. Studies have revealed that NK-92 cells modified with an anti-CD33 CAR are more sensitive and effective than CAR-T cells in killing CD33-positive leukemic cells. These genetically engineered NK cells also showed less cytotoxicity than unmodified NK-92 cells against the human Acute Myeloid Leukemic cell line HL-60 and have proved to be safe in patients with relapsed or refractory Acute Myeloid Leukemia (Albinger et al. 2022; R. Huang et al. 2025a, b; Li et al. 2025a; Rafiq et al. 2015; Włodarczyk and Pyrzynska 2022).

Another study on a mouse model with a B-cell lymphoma, CD19-CAR-NK-92 cells, was able to stop leukemic cell progression, proving that genetically engineered NK-92 cells can provide a strong and specifically targeted anti-cancer therapy (Romanski et al. 2016). In another study, researchers compared CAR-NK cells made from adult peripheral blood and umbilical cord blood against CD19-positive leukemia. They found that CAR-NK cells from peripheral blood were much more effective at eliminating leukemic cells than those derived from umbilical cord blood (Herrera et al. 2019).

Researchers have created CAR-NK cells genetically designed to target FLT3, an overproduced protein in B-ALL. When these genetically engineered NK cells were given to those B-ALL mice, interestingly, those cells stopped the progression of B-ALL, which the unmodified NK-92 cells couldn’t do. These results suggest that FLT3-CAR-NK cells could offer a powerful, new, promising way to treat B-ALL (Oelsner et al. 2019). CD7-CAR-NK-92 cells have also shown a strong and targeted anti-tumor activity against T-cell acute lymphoblastic leukemia, on cell lines and on mouse experimental models. This effect was likely driven by their ability to release higher amounts of granzyme B and IFN-γ, supporting the idea that CAR-NK therapy could also be a promising approach for treating T-cell-related cancers (You et al. 2019).

#### Lymphoma and myeloma

CAR-NK cells have also shown promising results in lymphoma and multiple myeloma treatment. In lymphoma research, CD19-CAR memory-like NK cells revealed strong anti-cancer properties. This was found to be associated with high IFN-γ production and selective destruction of malignant cells in vitro (Bastrich et al. 2025; Jørgensen et al. 2025; Sadurski et al. 2025). When CAR-modified memory-like (ML) NK cells were developed from patients with lymphoma, they were found to reduce lymphoma growth and extend survival in animals, and were not just limited to improving responses against their targeted cancerous cells (Gang et al. 2020).

In multiple myeloma, CAR-NK cells targeting CS1 (CD319), which is widely expressed on multiple myeloma cells, efficiently attacked and destroyed CS1-positive cancer cells and also boosted IFN-γ production and secretion in the tumor microenvironment, thereby slowing tumor-cell progression and proliferation. Additionally, it showed a significant prolongation of the survival rates in xenograft mouse experimental models ^[1]^. In addition to CD319, CD38-CAR engineered into NK-92 cells exhibited potent cytotoxic activity against primary myeloma cells derived from patients. These genetically engineered NK cells were particularly sensitive and selective, as they only attack CD38-positive tumors while leaving CD38-deficient cells untouched, and they successfully destroyed all CD38-positive multiple myeloma cells in bone marrow samples from multiple myeloma patients (Liao et al. 2023; Poels et al. 2021).

### Applications of CAR-NK cells in solid tumors: mechanistic insights and therapeutic outcomes

There are several explanations for why CAR-NK cells have a limited lifespan in solid tumors. For starters, NK cells are already different from longer-lived lymphocytes like T cells in that they typically have a quicker turnover rate in vivo and a limited lifespan. Add to that the hypoxia, deprivation of nutrients, and exposure to immunosuppressive substances, including prostaglandin E2 (PGE2) and adenosine, which worsen the problem in the tumor setting by encouraging NK cell death and malfunction. Furthermore, the balance between activating and inhibiting signals controls the NK cells' cytotoxicity and activation, so prolonged activation can result in a state marked by a decline in survival and functionality, like that of T cells (Balkhi et al. 2025; S. Li et al. 2025a, b).

The treatment of solid tumors represents a far greater challenge in comparison to hematological malignancies. Unlike blood malignancies, solid malignancies have a confined environment to grow and develop, known as the tumor microenvironment (TME). TME not only isolates the tumor from the rest of the body but also plays a crucial role in suppressing the anti-tumor immune responses against cancer cells, creating a powerful shield that is particularly difficult to evade and supports its survival and growth. These barriers arise from complex interactions between the TME and the surrounding immune components (Esmaeilzadeh et al. 2024).

Traditional therapies have shown limited effectiveness against solid malignancies. An analysis of 40 clinical trials conducted through 2015 to 2019, and the evaluation of 36 FDA-approved drugs for solid tumors, has reported modest outcomes, with an overall response rate of 52.5%, a progression-free survival rate of about 32.5%, and an overall survival rate of just 5% (Kaur et al. 2023; Zafar et al. 2025).

CAR-T cell immunotherapy has achieved remarkable success in treating various hematologic malignancies, but up to now, its impact on metastatic solid tumors is still limited due to significant challenges. While CAR-NK cells offer several advantages: providing strong cytotoxic capacity, the ability to recognize and target cancer cells without MHC restriction, natural infiltration into tumor tissues, relatively easy obtaining and preparing, and having a much lower risk of severe side effects such as cytokine release syndrome (CRS), graft-versus-host disease (GvHD), and neurotoxicity, which are common in CAR-T cells. These advantages collectively make CAR-NK an excellent alternative treatment in solid tumors.

Thanks to these properties, genetically engineered CAR-NK cells are being significantly explored as a promising therapeutic option for solid tumors (Dagar et al. 2023; Du et al. 2025; X. Zhang et al. 2022a, b; Zugasti et al. 2025). Preclinical studies have already tested CAR-NK cells in a wide range of cancers, including breast cancers and TNBC, ovarian cancers, pancreatic and colorectal cancers, liver (hepatocellular carcinoma), glioblastomas, Neuroblastomas, gastric and Esophageal squamous cell carcinomas, and head and neck squamous cell carcinomas (HNSCC) (R. Kong et al. 2025a, b; Li et al. 2022; Luo et al. 2026) For instance, several studies have demonstrated that genetically engineered CAR-NK cells targeting EGFRvIII were effective in xenograft models, successfully attacking glioblastoma cells that secrete CXCL12/SDF-1α as well as EpCAM-positive colorectal cancer cells following intravenous infusion (Han et al. 2015; Li et al. 2025b; Martins et al. 2024).

Despite the inspiring preclinical findings, clinical evidence for the use of CAR-NK cells in solid tumors remains very limited. Notably, three phase I/II trials in China (NCT03940820, NCT03941457, NCT03931720) evaluated allogeneic ROBO-1-CAR-NK-92 cell therapy in patients with pancreatic ductal adenocarcinoma (PDAC) and other ROBO-1–expressing solid tumors. The outcomes from these studies suggested that CAR-NK therapy could be a feasible approach beyond hematologic cancers. In addition, a phase II trial in the United States (NCT04847466) is currently investigating PD-L1-CAR-NK cells in combination with the IL-15 agonist N-803 and the immune checkpoint inhibitor pembrolizumab. These trials highlight the interest in integrating CAR-NK therapy with other antitumor agents, hoping to reshape the immunosuppressive tumor microenvironment (TME) to better control the aggressiveness and metastatic potential of resistant solid tumors (T. Li et al. 2024b; L. Zhang et al. 2022a, b).

#### Breast cancer

Breast cancer is the most common cancer among women all over the world and the Leading cause of cancer-related deaths among women. Early-stage clinical trials assessing CAR-NK cell therapy for breast cancer still exist, with various studies already recruiting or active, but still too early to make any conclusions regarding the safety and efficacy profile of these genetically engineered therapies.

The epidermal growth factor receptor (EGFR), as a canonical tyrosine kinase receptor, is yet at the center stage of targeted cancer therapy (Ahmed et al. 2026; Rana et al. 2025). Recently, some studies have revealed evidence about EGFR-specific CAR-NK-92 cells and primary NK cells to stimulate strong anti-tumor activities in vitro against MDA-MB-231, MDA-MB-468, and MCF-7 lines of breast cancer cells. EGFR-specific CAR-NK-92 cells demonstrated high-level cytotoxicity and release of IFN-γ, proving to have a better therapeutic potency than primary NK cells. Direct intratumoral delivery of either EGFR-CAR-NK-92 cells or oHSV-1 reduces the xenograft breast tumor outgrowths in xenograft models of breast cancer; the combination of these two therapeutics resulted in better cancer elimination and improved survival rates among mice (Chen et al. 2016; J. Li et al. 2024a), compared to when EGFR-CAR-NK-92 is given alone (Y. Liu et al. 2020).

#### Ovarian cancer

Ovarian Cancer is the third most common cancer and the most lethal gynecological malignancy among women around the world. In the context of the specificity and efficacy of CAR-NK cells for ovarian Cancer, an early-phase I randomized controlled trial is ongoing to test the effectiveness of anti-mesothelin CAR NK cells on epithelial ovarian cancer patients with more than 50% mesothelin after standard chemotherapy (NCT03692637). Another trial is being conducted on various types of solid cancers, including epithelial ovarian cancer, to evaluate the safety and efficacy of anti-NKG2DL CAR NK cells against those cancers (NCT03415000). Recently, another clinical trial is being performed aiming to evaluate the efficacy and safety of Anti-CLDN6 CAR-NK cells in CLDN6-positive advanced solid malignancies, including ovarian cancer (NCT05410717). Most of the studies conducted have reported the safety of using CAR-NK cells as cancer immunotherapies; despite this, there was a reported clinical trial that was held to evaluate the safety of anti-α FR CAR-NK cells was early terminated due to its toxicity effect (NCT00652899) (Dash et al. 2024; Esmaeilzadeh et al. 2024).

Furthermore, it has been indicated that anti-mesothelin CAR-NK cells can selectively destroy mesothelin-positive ovarian cancer cells, such as OVCAR-3 and SK-OV-3, while not reacting against mesothelin-negative cells, such as SK-HEP-1 (An et al. 2025; Cao et al. 2020). More importantly, anti-mesothelin CAR-¬NK cells have efficiently eradicated ovarian cancer cells in subcutaneous and intraperitoneal tumor models, indicating that mesothelin and αFR could be good targets for ovarian cancer CAR-NK targeted therapy (Guo et al. 2024).

Other findings have recently suggested that co-expression of chemokine receptor CXCR1 and CAR can be an innovative strategy improving the efficacy of NK cells against human ovarian tumors by increasing the infiltration of immune cells into tumor tissue. Studies have indicated that administration of CAR-NK cells expressing CXCR1 targeting NKG2D ligands has demonstrated a better migration of immune cells to tumor cells in vivo and in xenograft models, and CAR-NK cells that have not been negatively affected by CXCR1 expression have boosted anti-tumor reactions in xenograft models (Jin et al. 2019; Ng et al. 2020).

#### Lung cancer

Various types of CAR-NK therapies have been investigated in lung cancer treatment, including two approved clinical trials. To reverse the immunosuppressive effect of PD1, a chimeric co-stimulatory transition receptor (CCCR) consisting of PD1 extracellular domain, NKG2D transmembrane and cytoplasmic domain, and NKG2D cytoplasmic domain 4–1BB was applied. The mechanism is attributed to the fact that CCCR can convert negative PD1 signals into activation signals. In this context, CCCR-NK92 cells in a lung cancer xenograft model demonstrated significant inhibition of tumor growth (Lu et al. 2020).

DLL3 is a highly tumor-selective cell surface protein that is overexpressed in high-grade neuroendocrine lung tumors, including SCLC. Liu et al. engineered DLL3-specific NK-92 cells that induce tumor regression in an H446-derived lung metastatic tumor model with a favorable safety profile (Liu et al. 2022). Moreover, an ongoing clinical trial has started this approach for treating SCLC patients (NCT05507593).

Mesenchymal–epithelial transition factor (C-Met) has been exhibited as a prominent therapeutic target for treating lung adenocarcinoma (LUAD). In vitro and vivo, the anti-c-Met scFv were employed with different combinations (CC1, CC2, CC3, CC4) (Peng et al. 2023)*.* This tumor-inhibitory effect is conferred by the DAP10 designed in the CAR structure, stimulating the NK cell activation, and CCN4, containing DAP10 co-stimulator, exerted the strongest cytotoxicity compared with other CAR-NK cells. Moreover, CCN4 cells also demonstrated a significant tumor-inhibitory effect on xenograft tumor growth. Accordingly, this study suggests that CCN4-based immunotherapy may represent a promising strategy for the treatment of c-Met-positive LUAD, and DAP10 is a potent stimulator in the CAR structure for NK cell activation.

In vitro, the dtCAR-NK92 cells exhibited improved NK cell cytotoxicity and produced more cytokines than NK92 cells when they were co-cultured with human lung cancer H1299 cells (Zhi et al. 2025). Notably, the dtCAR-NK92 cell therapy might elicit clearance of H1299 cells by proptosis and could inhibit tumor growth in H1299 cells, which provides a novel immunotherapeutic strategy for engineering CAR-NK92 cells to treat human lung cancer.

Guevara Lopez et al. focused particularly on the CD56^bright^ NK-cell subset, which is known for its strong cytokine-producing capacity, investigating cytokine-induced memory-like (CIML) NK cells as a therapeutic strategy in non-small cell lung cancer (NSCLC). CIML-NK cells targeting DLL3 significantly outperformed conventional IL-2-activated NK cells in killing NSCLC cell lines, and also demonstrated that the CD56^bright^ subset expanded markedly during CIML differentiation (Liu et al. 2022) and the 3D spheroids, showing higher IFN-γ secretion and CD107a degranulation.

NK-92MI cells carrying an anti-B7-H3 CAR effectively restricted the growth of non-small cell lung cancer, with significant prolonged survival time compared to unmodified NK-92MI cells (Yang et al. 2020). This may be because perforin/granzyme B secretions and CD107a expression were notably elevated in anti-B7-H3 CAR-NK-92MI cells. In another study, Chambers et al. designed a CAR-NK targeting the CD73 adenosine axis; this approach induces tumor stasis, in addition to impairing the adenosine metabolism driven by hypoxic ATP uptake by cancer cells in an NSCLC model, and promotes NK cell infiltration into CD73 tumors, and enhances intratumoral activation (Chambers et al. 2022).

#### Glioblastoma

In vitro and in orthotopic GBM xenograft models, Zhang et al. designed a novel CAR-NK cell line (HER-2)-specific NK-92/5.28.z against GBM cells that demonstrated a potent anti-tumor effect (Zhang et al. 2014), and Murakami et al. designed a novel CAR-NK cell line (CAR-KHYG-1) for tumors expressing epidermal growth factor receptor variant III (EGFRvIII) that EvCAR-KHYG-1 inhibits GBM cell growth through apoptosis (Murakami et al. 2018). Then, a further mechanistic study suggests the long-lasting anti-tumor response is attributed to that NK-92/5.28.z triggers the release of tumor antigens, which, in turn, activate immune cells, and then high levels of pro-inflammatory cytokines are secreted (Myers and Miller 2021). Combining NK-92/5.28.z with systemic anti-PD-1 reverted the immunosuppressive tumor microenvironment with increased T and NKT cell infiltration, induced robust anti-tumor response, and long-term survival (Strassheimer et al. 2025). This approach opens doors for more combinational rationales, especially immunosuppressive TME, and in resistance either to immune checkpoint inhibitors, or CAR NK therapy.

CSPG4 (chondroitin sulfate proteoglycan 4), a tumor-associated antigen that is overexpressed in GBM, is associated with tumor proliferation and metastasis and accounts for a promising therapeutic target. In a preclinical study, CSPG4-targeted CAR-NK cells selectively recognized and lysed CSPG4-positive GBM cells, significantly suppressing tumor growth compared to control NK cells (Xiong et al. 2025). Their mechanism of cytotoxicity was mediated through specific CSPG4 antigen engagement. These findings provide a robust translational foundation for advancing CSPG4-targeted CAR-NK cell therapy, addressing the urgent need for effective treatments in GBM management.

Dual targeting (e.g., EGFR and EGFRvIII) is an innovative approach has shown superior anti-tumor effects through mitigating antigen escape. In xenografts, DAP12.CD3.CAR-NK-YTS cells targeting EGFRvIII prolonged survival and inhibited tumor growth (Lanier 2009). Genßler et al. developed a dual-targeting CAR-NK cell ( EGFR and EGFRvIII), and CD28 and CD3ζ are the costimulatory domains. In the xenograft model, dual-target-specific CAR-NK cells can effectively diminish tumor growth and prolong the survival time of mice (Genßler et al. 2016). Additionally, Muller et al. constructed CAR-NK cells targeting EGFRvIII, and DAP12 is the costimulatory domain. In the xenograft model, CAR-NK cells also diminished tumor growth and prolonged the survival of mice. Moreover, Tsutomu et al. developed CAR-NK cells targeting EGFRvIII, and CD28, 4-1BB, and CD3ζ are the costimulatory domains. In the xenograft model, CAR-NK cells targeting EGFRvIII can effectively diminish tumor growth (Nakazawa et al. 2020). Further clinical trials should integrate multiple targeting strategies and TME reprogramming to enhance CAR-NK therapy’s efficacy in GBM.

#### Neuroblastoma

Neuroblastoma, which is a malignant tumor of the sympathetic nervous system, arises from neural crest cells that form the adrenal medulla and sympathetic ganglia. It is the most common extracranial solid tumor in children and the most common cancer in infants (Ponzoni et al. 2022).

Studies have found that anti-Ganglioside or anti-GD2 CAR-NK cells attack GD2-positive neuroblastoma and can significantly induce higher cytotoxic effect towards Neuroblastoma cells, leading to suppressing its progression and development, and repressing tumor development in mice (Jünemann et al. 2025; Locatelli et al. 2025; Philippova et al. 2024; Seidel et al. 2015).

In addition, anti-GD2-CAR-NK cells have the ability to be introduced into 3D tumor spheroids, resulting in a reduced growth rate and progression of the Neuroblastoma, which has been proven by live-cell imaging analysis. It’s also been indicated that the combination of redirected NK cells and either histone deacetylase inhibitors (HDACi) or programmed cell death protein 1 (PD-1)/programmed cell death ligand 1 (PD-L1) blocker can be more effective against cancer (Tirapelle et al. 2024; Zuo et al. 2023).

#### Liver cancer

Liver cancer is one of the most common types of cancers in the world, with about 905,700 new cases and about 830,200 deaths every year. Hepatocellular carcinoma is the most common type, accounting for 93% of primary liver cancer. GPC3 is a protein that is highly expressed in hepatocellular carcinoma. Studies have indicated that CAR-NK cells are suggested as a potential immunotherapy for hepatocellular carcinoma. anti-GPC3-CAR-NK-92 cells attack hepatocellular carcinoma cells, causing a major cytotoxic effect against tumor cells in vitro; it also stimulates an anti-tumor effect on hepatocellular carcinoma xenograft models as well (Lin et al. 2024; Park et al. 2026).

Anti-GPC3-CAR-NK-92 cells were found to have a suppressing effect on tumor development and enhance tumor apoptosis in GPC3-positive hepatocellular carcinoma xenograft models. Also, it has been noted that hepatocellular carcinoma patients who have high serum levels of sPD-L1 show higher suppression activity of anti-GPC3-CAR-NK cells against cancer cells. To encounter this hurdle, a high-affinity sPD-L1 variant (L3C7c-Fc) has been integrated to GPC3-CAR-NK cells to overcome the suppressive effect of sPD-L1 by neutralizing the inhibitory effect of sPD-L1(Chen et al. 2023). This combined strategy has shown promising prospects in enhancing the therapeutic efficacy for hepatocellular carcinoma.

TGF-β is used to suppress NK cell function. Wang et al. have genetically engineered NK-92 cells to express a TN chimeric receptor, which is composed of an extracellular and transmembrane chimeric receptor with the TGF-β type II receptor. CAR-NK-92 cells with TN-expressing can resist the suppressive signals from TGF-β, and they also have increased killing capacity against hepatocellular carcinomic cells in vitro. TN-expressing CAR-NK-92 cells also showed an effect on suppressing tumor progression of hepatocellular carcinoma on xenograft models, suggesting that the TN-expressing CAR-NK-¬92 cells have a promising therapeutic effect on hepatocellular carcinoma (Wang et al. 2017).

c-MET is a protein present in the epidermal, endothelial, neurons, hepatocytes, and hematopoietic cells. c-MET has been considered as an oncogene in hepatocellular carcinoma, which can promote, maintain, or even worsen the tumorigenic process and the disease progression. Researchers have developed CAR-NK cells specific for c-MET. c-MET-CAR-NK cells have shown cytotoxic effects on HepG2 cells with high endogenous c-MET levels in vitro, which is why anti-c-MET-CAR-NK cells have promising results to be used as immunotherapy for human liver cancer cells (Liu et al. 2019; Peng et al. 2023).

#### Colorectal cancer

High expression of EpCAM (CD326) is prevalent in solid tumors of epithelial origin, including CRC. Preclinical research is developing targeting for EpCAM-positive cells with immune-mediated therapeutic intervention(W. Li et al. 2021a, b; Schnalzger et al. 2019). The effect of CAR-NK-92 targeting EpCAM was examined in the CRC line HCT-8-Luc. CAR NK-92 cells significantly inhibited tumor growth when compared to the control NK-92 cell line (Xiao et al. 2019). Moreover, preclinical studies are crucial for recreating a distinct tumor phenotype. Providing long-term extracellular 3D matrix growth of gastrointestinal stem cells is also essential. In this context, Schnalzger et al. assessed EpCAM CAR NK-92 and FRIZZLED CAR NK-92 effectiveness in patient-derived colorectal cancer organoids in CRC (Schnalzger et al. 2019b).

Mucin 1 (MUC-1),a highly glycosylated protein that is highly expressed in CRC cells, and human epidermal growth factor receptor 2 (HER2) are two important targets for cell-mediated therapy. A notable enhanced cytotoxicity against HER2-positive targets both in vivo and in vitro in the modified subpopulation of NK-92, which expresses functional endogenous CD16 and is further modified by conjugation with Trastuzumab, an anti-HER2 antibody (Gao et al. 2021; H.-K. Li et al. 2021a, b). An ongoing phase I/II clinical trial (NCT02839954) is investigating the anti-MUC1 CAR NK cells for patients with solid tumors, such as CRC (Hossian et al. 2022). An ongoing clinical trial (NCT04319757),is investigating the anti-HER2 CAR NK cells for patients with solid tumors, such as CRC (Ghazvinian et al. 2023).

Carcinoembryonic antigen** (**CEA), a fetal glycoprotein and tumor antigen, is largely expressed in CRC tissues that can be used to guide targeted therapy (NCT02349724) (Zhang et al. 2017). In a preclinical study, anti-CEA CAR NK-92MI eradicated CEA-expressing tumor cells at high and moderate levels (Shiozawa et al. 2018). These anti-CEA-modified NK cells may be a secondary rescue line of treatment for CRC that is resistant to other forms of therapy, since chemotherapy commonly upregulates the CEA. Despite these promising efficacy results, some concerns have been raised about safety; in turn, further investigations are warranted. However, these raised concerns, a preclinical study exerted a minimal off-target activity upon treating CEA-CAR-NK-92 cells against CRC in colorectal cell lines and MCTS models. The CEA-targeting CAR-NK cell therapy combining precision and efficacy in a tailored approach could offer a promising modality for CRC treatment (Franzén et al. 2024).

Regulatory T (Treg) cells expressing CD25 are a pivotal immunosuppressive element in colon cancers (Aristin Revilla et al. 2022). Accordingly, Dehbashi et al. developed a novel CAR NK cell targeting CD25 to overcome this immune escape mechanism and to specifically detect and lyse target cells with CD25 expression, potentially addressing the resistance by Treg cells (Dehbashi et al. 2021). To validate and optimize this approach, future investigations are warranted.

In a pilot clinical trial (NCT03415100), three resistant metastatic CRC patients were successfully treated with a local infusion of NKG2D CAR NK (one in an autologous context and two in a haploidentical environment). Two patients in particular saw a reduction in the amount of cancer cells in their ascites fluid, while another patient’s liver metastasis showed a full metabolic response. Another clinical trial (NCT05213195), evaluating the outcomes of intraperitoneal and intravenous NKG2D-CAR NK infusion in patients with refractory metastatic CRC, will also provide additional insightful results in this regard.

#### Pancreatic and gastric cancers

Pancreatic cancer is the 4th most common cancer-related death in the USA because of its aggressiveness and invasiveness. Mesothelin is highly expressed on the surface of several tumor types, including pancreatic cancer cells. Researchers have developed CAR-NK cells, which specifically target MSLN-expressing cells. MSLN-CAR-NK cells derived from the NK-92 cell line were found to mediate IFN-γ and granzyme B, and can specifically lyse the pancreatic cancer cells in vitro. MSLN-CAR-NK cells have also shown good tumor reduction in a pancreatic cancer xenograft model in vivo. The combination of MSLN-CAR-NK cells with the STING agonist cGAMP has significantly reduced the tumor growth and development, increasing the survival of the xenografts. These findings introduce possibilities of treating pancreatic cancer with CAR-NK cells (An et al. 2025; Jung et al. 2026).

**Fig. 3:**
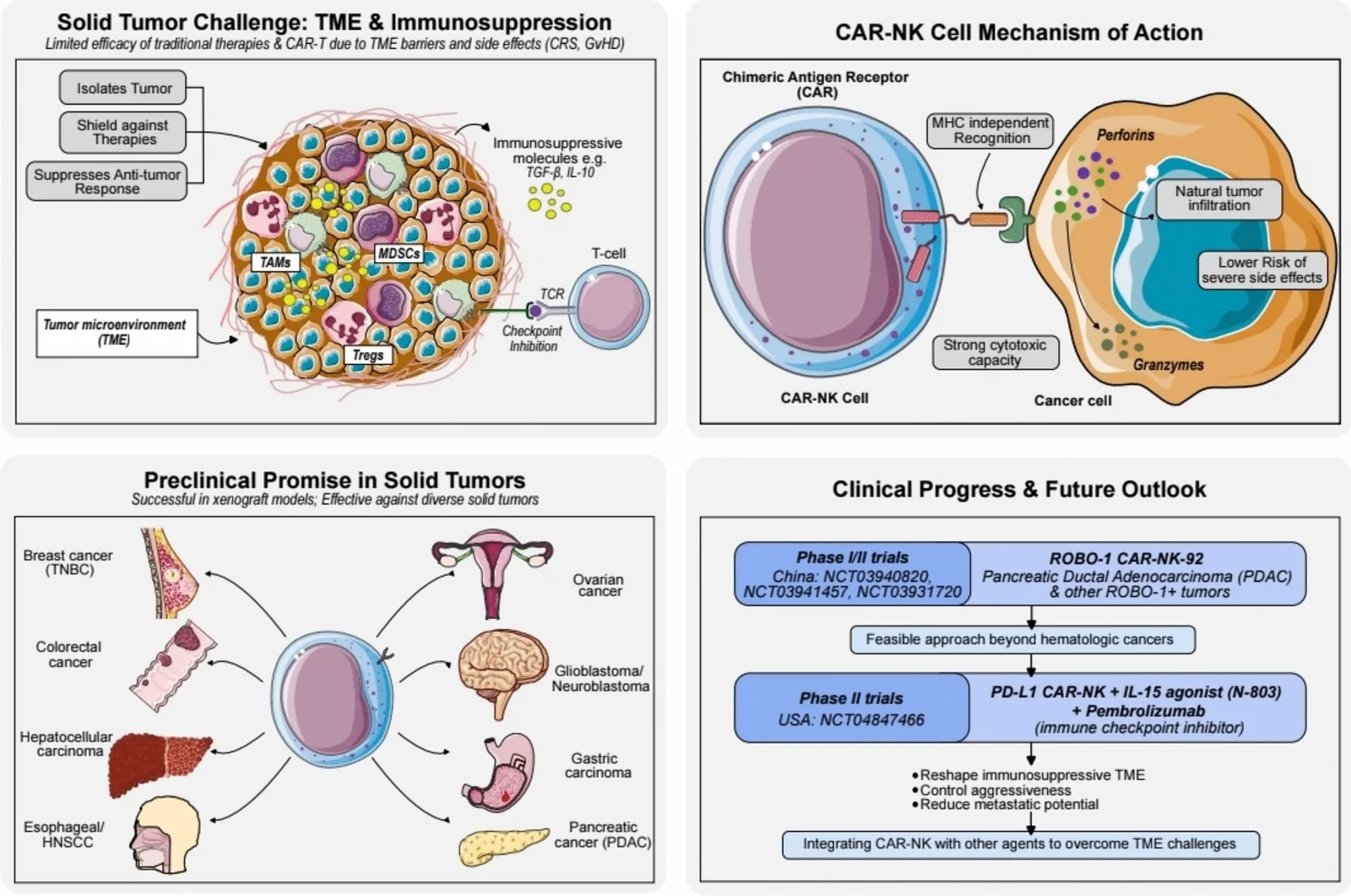
Graphical representation of the CAR-NK progress and overall summary. Icons have been adapted and recreated from Servier Medical Art (https://smart.servier.com/), licensed under CC BY 4.0 (https://creativecommons.org/licenses/by/4.0/)

**Fig. 4:**
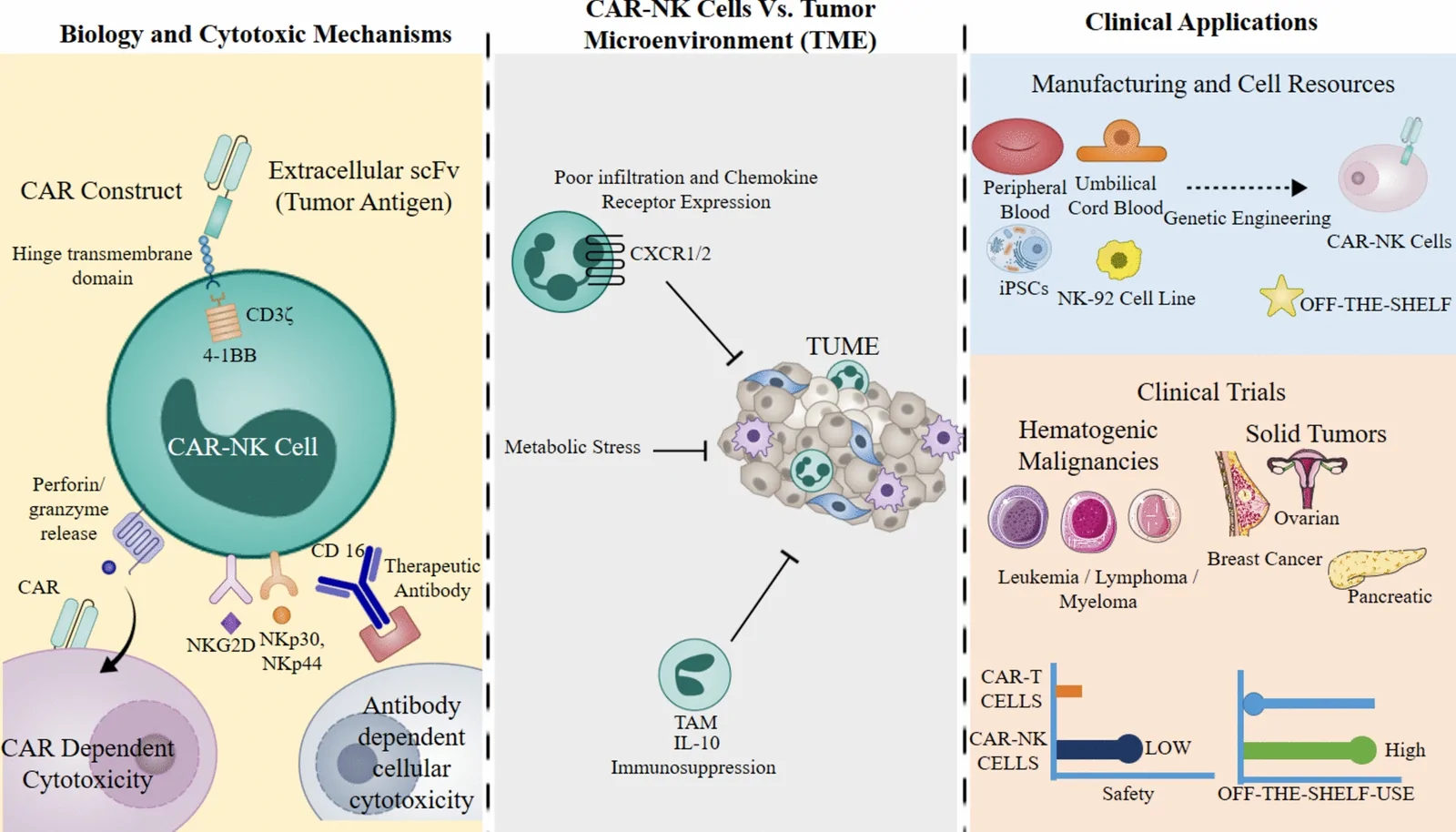
Mechanistic overview of CAR-NK cell therapy, interactions with the tumor microenvironment (TME), and current clinical translation. **(A)** CAR-NK cell biology and cytotoxic mechanisms: CAR-NK cells execute multi-modal anti-tumor responses through three distinct pathways: *CAR-Dependent Cytotoxicity:* Directed recognition of target tumor-associated antigens via single-chain variable fragments (scFv) triggering intracellular signaling cascades (CD3ζ, 4-1BB, or NK-native adapters such as DAP10/DAP12) and inducing targeted perforin and granzyme secretion. *Natural NK Cytotoxicity:* Innate recognition mediated by activating receptors (e.g., NKG2D, NKp30, NKp44, 2B4) engaging stress ligands, allowing for effective killing under "missing-self" conditions or during tumor antigen loss. *Antibody-Dependent Cellular Cytotoxicity (ADCC):* Engagement of the Fc region of therapeutic monoclonal antibodies via surface CD16 (FcγRIIIa) receptors. **(B)** CAR-NK cell interactions with the solid tumor microenvironment (TME): Key obstacles hindering CAR-NK efficacy in solid tumors and corresponding counter-engineering strategies include: *Infiltration Barriers:* Low expression of homing receptors limits trafficking; engineered co-expression of chemokine receptors (e.g., CXCR1, CXCR2) restores targeted migration toward tumor gradients. *Immunosuppressive Cellular/Cytokine Networks:* TME factors including regulatory T cells (Tregs), myeloid-derived suppressor cells (MDSCs), tumor-associated macrophages (TAMs), and TGF-β/IL-10 suppress cytotoxicity; arming CAR-NK cells with autocrine IL-15 or knocking out inhibitory receptors (e.g., NKG2A, TIGIT, PD-1) reverses exhaustion. *Metabolic Stress & Decoy Receptors:* Hypoxia and metabolic stress impair persistence; expression of dominant-negative TGF-β receptors protects NK cells from immunosuppressive TME signaling. (**C**) Manufacturing Sources and Clinical Translation: *Cell Sources & Off-the-Shelf Potential:* CAR-NK cells are derived from allogeneic sources including peripheral blood (PB), umbilical cord blood (UCB), induced pluripotent stem cells (iPSCs), and established cell lines (NK-92), enabling scalable, "off-the-shelf" manufacturing without strict HLA matching. *Clinical Outcomes in Hematologic vs. Solid Tumors:* High response rates and complete remissions have been demonstrated in hematologic malignancies (e.g., B-ALL, DLBCL, Multiple Myeloma targeting CD19 and BCMA) with favorable safety profiles (low incidence of CRS and neurotoxicity, no GvHD). Active exploration in solid tumors (targeting HER2, EGFRvIII, ROBO-1, Mesothelin) focuses on overcoming TME resistance to achieve durable anti-tumor responses

HER2 protein is expressed on a subtype of gastric cancer cells. Researchers have engineered CAR-NK-92 cells, which are called HER2-CAR-NK cells. HER2-CAR-NK cells can selectively target HER2-positive gastric cancer cells and produce a high level of cytokines to destroy the cells. suggesting a promising approach for treating gastric cancers (Zhu et al. 2025). Just like pancreatic carcinomas, MSLN is also highly expressed on gastric cancer cells. Researchers have engineered anti-MSLN-CAR-NK cells that target MSLN-expressing cells in patients with gastric cancer. These cells have selectively targeted and destroyed MSLN-positive gastric cancer cells, without attacking MSLN-negative cells, which is confirmed by in vitro studies indicating their promising therapeutic potential for gastric cancer immunotherapy (An et al. 2025; Cao et al. 2021) (Fig. 3,4).

## Limitations of CAR-NK cell therapy and strategies and future perspectives to overcome

### Biological and technical challenges of CAR-NK therapy

Despite CAR-NK exhibiting promising features, several significant limitations exist, hindering their successful application in treating solid tumors. Many of these limitations are also common with CAR-T therapy limitations, such as poor tumor infiltration and detrimental interactions with the TME. Moreover, NK cells have specific limitations unique to their biology and function. Clinical evidence suggests that the presence of activated NK cells infiltrating solid tumors correlates with positive prognostic outcomes (Tarannum et al. 2022). To enhance NK cell migration, various strategies have been tested. Genetic engineering CAR-NK cells to express chemokine receptors like CXCR4, CXCR1, or CXCR2 has resulted in increased migration toward corresponding chemokine gradients and enhanced in vivo efficacy in various cancer models (Ng et al. 2020). An alternative strategy involves the use of an NK cell-recruiting protein-conjugated antibody that releases a chemokine to create a gradient that enhances NK migration toward the tumor in vivo (Page et al. 2024).

One of the main limitations of CAR-NK cell application is the resistance of NK cells to viral transduction compared to T cells. This results in variable transduction efficiency, which represents a significant limitation for controlled clinical trials. The use of γ-retroviruses also implies a higher risk of insertional mutagenesis compared to lentivirus vectors. Various strategies have been explored to optimize a lentiviral transduction protocol for NK cells, including exposure to cytokine combinations or pretreatment with inhibitors such as the TBK1 inhibitor BX795 (Allan et al. 2021). These studies have demonstrated the possibility of increasing lentivirus transduction efficiency in human primary NK cells to 25–40%; therefore, further research and innovative solutions are needed to optimize this process in NK cells (Balkhi et al. 2025; Zugasti et al. 2025).

The future of CAR-NK therapy is promising with breakthrough advancements, based on the promising early results. The critical area of ongoing research on CAR-NK therapy is to enhance the persistence and expansion of NK cells in vivo, in turn, improving CAR- NK cells' therapeutic efficacy. Strategies are incorporating cytokine support to sustain NK cell activity, gene editing techniques to knock out inhibitory receptors, combining different therapy modalities, and developing various CAR designs that can boost NK cell potency (Balkhi et al. 2025; Tarannum et al. 2022).

### Tumor microenvironment challenges and strategies to overcome

The application of CAR-NK cells in solid tumors faces challenges related to trafficking toward the tumor. NK cells are often limited in their presence within several solid tumors, indicating difficulties in their ability to reach, infiltrate, and persist within the TME. Additionally, infiltrating NK cells in solid tumors often display a dysfunctional phenotype, partly due to detrimental factors such as hypoxia and soluble inhibitory factors present in the TME. The short half-life and short-term anti-cancer activity of NK cells also become even more crucial in the setting of solid tumors (Peng et al. 2024).

However, the TME remains a major obstacle, as tumors can release soluble factors, such as soluble NKG2D ligands (sNKG2D-L), which dampen the cytotoxic function of NK cells by downregulating their activating receptors (C. Liu et al. 2026b, a). To address this, advanced engineering strategies are being developed. These include creating membrane-anchored ectopic receptors to enhance tumor recognition, cytotoxicity, and TME resistance (Lei et al. 2026; Ma et al. 2025; L. Zhang et al. 2025a, b). A key strategy involves protecting CAR-NK cells from the immunosuppressive tumor microenvironment (TME). This can be achieved by engineering them to secrete cytokines like IL-15 to enhance their survival and activity, or by modifying them to express a dominant-negative TGF-β receptor to block this inhibitory signaling pathway (“CAR T Cells with a Dominant-Negative TGFβ Receptor Are Safe and Feasible,” 2022; Carton and Spiller 2025).

Furthermore, knocking out inhibitory receptors like NKG2A on CAR-NK cells can make them resistant to TME-suppressive signals, an approach shown to boost anti-tumor activity in preclinical models (Guo et al. 2025; Islam et al. 2025). Other design considerations involve genetically modifying CAR-NK cells to protect them from inhibitory metabolites, block immune checkpoint proteins like PD-1 or TIGIT, and equip them with chemokine receptors to improve tumor infiltration. In summary, genetic modification to express a CAR can counteract inhibitory signals, thereby specifically enhancing the NK cells' ability to kill target cells (Bakhtiari et al. 2026).

TGF-β dampens various aspects of NK cell anti-tumor function. Therefore, pharmacological inhibition of TGF-β or engineering the TGF-β receptor on NK cells can potentially overcome this inhibition (Wang and Bi 2026). A clinical trial is evaluating the use of NK cells genetically depleted for the TGF-β receptor 2. NK anti-tumor function is also affected by extracellular adenosine. Strategies to prevent this include using ectonucleotidase or A2 receptor inhibitors or engineering lymphocytes to suppress the expression of adenosine receptors (Johns et al. 2026). CAR-NK can also benefit from the concomitant use of checkpoint inhibitors against targets like CTLA4, PD-1, NKG2A, TIGIT, and TIM3 (Wang and Bi 2026). Myeloid-derived suppressor cells (MDSCs) harbor potent immunosuppressive activity on NK cells, encountering CAR-NK cell cytotoxicity, in turn, promoting tumor growth. Accordingly, strategies have emerged to mitigate MDSCs' functionality, increasing CAR-NK cytotoxicity through depleting them and targeting reactive oxygen species (ROS) production or arginase activity, specific signaling pathways involved in MDSC-mediated suppression (Hosseinalizadeh et al. 2025; Joshi and Sharabi 2022).

### Strategies to enhance CAR-NK persistence and antitumor activity

A primary challenge is the short half-life of NK cells post-infusion. NK cells have a short half-life and exhibit poor persistence once transferred from ex vivo conditions to in vivo conditions. This aspect could potentially imply the necessity for multiple infusions (Saultz and Otegbeye 2023). Current evidence seems to indicate that multiple administrations of CAR-NK are not associated with serious adverse events, but these data come from small early-stage trials (Rana et al. 2025). Furthermore, this approach could affect the cost-effectiveness of CAR therapy. For example, in a phase 1 clinical trial on patients with refractory acute myeloid leukemia, patients could receive multiple administrations of CAR-NK92 cells without severe adverse events, and the cells became undetectable after one week of administration (Tang et al. 2018). Also, there is therefore an active exploration into reprogramming CAR-NK cells to possess memory or memory-like properties, or to arm them with IL-15, aiming for long-term tumor control (Peng et al. 2024).

Advanced genetic engineering allows the creation of NK cells that carry additional therapeutic agents like cytokines to boost their survival. For instance, CAR-NK cells engineered to express IL-15 demonstrate enhanced persistence and increased anti-tumor activity in vivo. Other innovative approaches include incorporating gene-editing tools to remove pro-apoptotic genes or checkpoint genes like PD-1 to enhance resistance to the TME and prevent exhaustion, creating so-called "armored" CAR-NK cells (Balkhi et al. 2025; T. Li et al. 2024a). Another promising option is harnessing cytokine-induced memory-like (CIML) NK cells, which are generated through ex vivo priming with IL-12, IL-15, and IL-18 and exhibit prolonged persistence and higher anti-cancer activity (Feng et al. 2025).

Additionally, Tumor antigen escape is a significant hurdle where tumors downregulate or lose the target antigen. To address this, research has focused on developing CAR-NK cells with dual or multiple antigen-targeting capabilities (Ramakrishna et al. 2020). By recognizing more than one antigen, these multi-specific CAR-NK cells can reduce the likelihood of tumor escape due to antigen loss. For instance, a bispecific CAR that targets both HER2 and MUC1 can engage different populations within a heterogeneous tumor mass (Qutub et al. 2026). Another example is bispecific CAR-NK cells, targeting both CD19 and B-cell maturation antigen (BCMA), which demonstrated strong cytotoxic activity against B-cell acute lymphoblastic leukemia (B-ALL) and multiple myeloma (MM) in vitro (Roex et al. 2022). Additionally, CD19-specific CAR-NK cells, targeting the hnCD16 receptor and an interleukin-15/interleukin-15 receptor fusion protein (IL-15RF), exhibited good antitumor potency, reducing the potential for tumor evasion (Rahnama and Bonifant 2022). Also, designing multiplexed CAR configurations targeting both BCMA and GPRC5D can effectively lyse BCMA-negative target cells and elicit durable responses in MM models (Yang et al. 2023). Another innovative approach has been developed, leveraging innate cell memory. This approach utilizes memory-like NK cells covered with CARs targeting neoepitopes derived from intracellular mutations such as NPM1c, a specific variant of the NPM1 gene associated with 30–35% acute myeloid leukemia (AML), resulting in potent and specific activity against AML (Dong et al. 2020). Collectively, these findings and implications illustrate how CAR design can incorporate multiple specificities to improve treatment plans.

Further approaches and strategies are warranted to increase the antitumor effects of CAR-NK cells. Modulate the immunosuppressive TME enriched with inhibitory cytokines, regulatory T cells (Tregs) and myeloid-derived suppressor cells (MDSCs), coinhibitory receptors expressed by tumor cells (such as PD-1, NKG2A and TIM-3) and their ligands, metabolic products such as prostaglandin E2 (PGE₂) and adenosine, and tumor-derived exosomes (TDEs) (Wang et al. 2024; Zhong and Liu 2024a, 2024b)**.** For instance**,** programmed cell death ligand 1 (PD-L1) CAR-engineered NK cells are used to overcome resistance and have shown potential findings, as these engineered cells present synergistic antitumor activity when combined with T cell-based immunotherapy, targeting and eliminating the resistant tumor cell populations that escape T cell killing by upregulating PD-L1 (Lee et al. 2021). The dual-targeting CAR NK approach can potentially overcome antigenic escape, in turn, improving treatment efficacy in solid cancers, paving the way for expanding this strategy in numerous cancers with immunosuppressive and challenging TMEs. Additionally, genetic modifications can prolong the survival time and increase NK cell proliferation of NK cells, thereby this could improve their persistence and antitumor activity. In addition, the quality and safety of CAR-NK cell modification have been enhanced by innovative techniques like transposons, electroporation, lipid nanoparticle (LNP) delivery, trogocytosis, and gene editing of clustered regularly interspaced short palindromic repeats–associated protein 9 (CRISPR-Cas9). Such techniques additionally create fresh prospects for treatments (Hu et al. 2025).

### Next-generation CAR-NK engineering and combination strategies

Most CAR-NK cell studies simply adapt CAR constructs designed for T cells and do not optimize them for NK cell signaling. An intriguing strategy was proposed to improve the replication and multiplication of CAR-NK cells, modifying them to express IL-15/IL-15Rα. As NK cells expressing ncCD16A were cultured together with different therapeutic mAbs and tumor types, they demonstrated higher levels of ADCC and cytokine production with a notable shift in the ADAM17 area of cleavage in CD16A, achieving cleavage resistance and avoiding the loss of CD16 (Schmidt et al. 2022; Xu et al. 2025). Optimizing the extracellular, transmembrane (TM), and intracellular signaling domains specifically for NK cells could lead to more effective therapies (Yi et al. 2025). For instance, using single-chain variable fragments (scFvs) derived from natural NK receptors like NKG2D or NCRs can leverage their broader tumor recognition to target multiple antigens and reduce immune escape. Utilizing TM domains from NK-activating receptors (e.g., DNAM-1, 2B4, NKG2D) rather than T-cell domains can improve signal transduction and yield higher cytotoxicity. Incorporating intracellular co-stimulatory domains native to NK cells, such as 2B4, DAP10, or DAP12, instead of or in addition to CD28 or 4-1BB, can also enhance anti-tumor efficacy (Lee et al. 2025).

Various sources for NK cells present different advantages and disadvantages. The immortalized cell line NK-92 offers unlimited expansion but requires irradiation before use, reducing persistence, and lacks CD16 expression (Klingemann 2025). Peripheral blood (PB) is a common source but requires apheresis from a donor. Umbilical cord blood (CB) is a rich source with a high percentage of NK cells and hematopoietic progenitor cells (HPCs), though these cells can show weaker cytotoxic activity. Hematopoietic stem cells (HSCs) offer the potential for standardized "off-the-shelf" therapies. Induced pluripotent stem cells (iPSCs) are particularly promising for generating a large number of homogeneous, gene-edited NK cells from a single clone, and iPSC-derived NK cells have been shown to exhibit potent cytotoxicity (Klingemann 2023; Lee et al. 2024; Von Werz et al. 2026).

Notably, CAR-NK cells can exert cytotoxicity through both CAR-dependent and natural mechanisms. This allows for the engineering of a CAR that triggers only a mild activation signal, minimizing off-target cytotoxicity against healthy tissues that express the antigen and enabling the targeting of a broader spectrum of antigens (Cianga et al. 2025). Identifying suitable tumor-specific antigens that are highly expressed on cancer cells with minimal expression on normal tissues is essential. Given tumor heterogeneity, developing multi-target CAR-NK cells may be beneficial to prevent antigen escape (Zhu et al. 2024). The tumor microenvironment significantly hinders immune cell function. Strategies to counteract this include targeting cancer-associated fibroblasts (CAFs) or modifying NK cells to resist immunosuppressive factors like TGF-β, for example, by reducing TGF-β receptor II expression. Tumor cells also evade immunity by up-regulating ligands for inhibitory receptors. Strategies to overcome this include knocking down inhibitory receptors like NKG2A on NK cells or combining therapy with checkpoint inhibitors (Hu and Huang 2022).

Combining CAR-NK cell therapy with chemotherapy or radiotherapy can create synergistic effects by increasing tumor antigen exposure and enhancing cancer cell sensitivity to immune killing. Additionally, the exploration of bispecific or multispecific CARs can also increase the likelihood of effective tumor eradication by facilitating the targeting of multiple tumor antigens simultaneously (J. Li et al. 2024b). Radiotherapy can also induce the expression of NKG2D ligands on tumor cells, enhancing NK cell activation. Combining CAR-NK cells with immune checkpoint inhibitors (ICIs) such as anti-PD-1/PD-L1 antibodies has been shown to synergistically enhance anti-tumor efficacy. Alternatively, using gene-editing techniques like CRISPR/Cas9 to disrupt checkpoint expression (e.g., PD-1) directly in CAR-NK cells represents a forward-looking strategy to enhance their function against solid tumors. Furthermore, sequential therapy using readily available "off-the-shelf" CAR-NK cells to reduce tumor burden first, followed by CAR-T cell infusion, could facilitate swift and lasting tumor reduction while potentially decreasing the likelihood of severe adverse effects like CRS (Guo et al. 2025; J. Li et al. 2024a).

## Conclusion

CAR-NK cell immunotherapy is a promising line of cellular oncology. It retains the targeting advantages of CAR technology while taking advantage of NK cells’ innate, multi-modal cytotoxic mechanisms and an inherently safer toxicity profile. Early clinical data from the cord blood-derived CD19 CAR-NK experience demonstrates both feasibility and favorable safety. Yet, translation into durable, broad-spectrum clinical benefit requires addressing four interdependent gaps.

First, CAR design must be NK-centric, not only from T-cell constructs. Optimizing extracellular scFv selection, transmembrane domains, and intracellular signaling (2B4/DAP10/DAP12) to align with NK biology will improve activation without premature depletion and exhaustion. Second, persistence and in vivo fitness are essential. Engineering strategies that provide autonomous survival signals (e.g., membrane-tethered IL-15), adopt cytokine-induced memory-like programming, or remove inhibitory checkpoints (TIGIT, NKG2A) can extend functionality while retaining safety. Third, overcoming the solid-tumor TME requires multimodal solutions, e.g., chemokine receptor engineering to enhance homing (CXCR1/CXCR4), dominant-negative TGF-β receptors or metabolic resistance modules, and rational combinations with ICIs, STING agonists, or localized modalities to remodel immune suppression. Fourth, manufacturing and regulatory harmonization are essential to scale. Standardized potency assays, optimized cryopreservation protocols, and safer gene-delivery platforms (transposon systems, high-efficiency CRISPR nucleases) will reduce cost and regulatory risk while mitigating insertional-mutagenesis concerns.

To be concise, CAR-NK therapy stands at an inflection point. Focused engineering to improve persistence and tumor targeting, paired with scalable, safe manufacturing and rigorously designed randomized trials, will determine whether CAR-NK cells evolve from a promising option to a standard tool against both hematologic and select solid malignancies. The analysis of preclinical and early clinical evidence contained in this review supports a strategic, modular roadmap for that advancement.

## Data Availability

No datasets were generated or analysed during the current study.

## Abbreviations

NK: Natural killer
ILC: Innate lymphoid cell
CAR: Chimeric antigen receptor
CAR-NK: Chimeric antigen receptor-natural killer
MHC: Major histocompatibility complex
MHC-I: Major histocompatibility complex class I
ITIMs: Immunoreceptor tyrosine inhibitory motifs
KIR: Killer-cell immunoglobulin-like receptor
FasL: Fas ligand
TRAIL: Tumor necrosis factor-related apoptosis-inducing ligand
IFN-γ: Interferon-gamma
TNF-α: Tumor necrosis factor-alpha
DC: Dendritic cells
ADCC: Antibody-dependent cellular cytotoxicity
AML: Acute myeloid leukemia
TME: Tumor microenvironment
IL-10: Interleukin-10
TGF-β: Transforming growth factor-beta
TAAs: Tumor-associated antigens
Tregs: Regulatory T cells
MDSCs: Myeloid-derived suppressor cells
CAFs: Cancer-associated fibroblasts
tDCs: Tolerogenic dendritic cells
NSCLC: Non-small cell lung carcinoma
IDO1: Indoleamine 2,3-Dioxygenase
TLR4: Toll-like receptor 4
iNOS: Inducible nitric oxide synthase
LDH5: Lactate dehydrogenase 5
ACT: Adoptive cell therapy
ICIs: Immune checkpoint inhibitors
irAEs: Immune-related adverse events
PD-1: Programmed cell death protein 1
PD-L1: Programmed cell death ligand 1
TRUCKs: T-cells redirected for universal cytokine killing
JAK/STAT: Janus kinase/signal transducer and activator of transcription
B-ALL: B-cell acute lymphoblastic leukemia
DLBCL: Diffuse large B-cell lymphoma
CRS: Cytokine release syndrome
GVHD: Graft-versus-host disease
TIGIT: T-cell immunoreceptor with Ig and ITIM domains
KLRC1: Killer cell lectin-like receptor C1
HLA: Human leukocyte antigen
iPSC: Induced pluripotent stem cell
PGE2: Prostaglandin E2
mTOR: Mechanistic target of rapamycin
Drp1: Dynamin-related Protein 1
DAP10: DNAX-activating Protein 10
DAP12: DNAX-activating Protein 12
GM-CSF: Granulocyte–macrophage colony-stimulating factor
CRISPR-Cas9: Clustered regularly interspaced short palindromic repeats–associated Protein 9
LNP: Lipid nanoparticle
CD16a: Cluster of differentiation 16a
ncCD16A: Non-cleavable CD16A
ADAM17: A disintegrin and metalloproteinase 17
NKG2D: Natural killer group 2D
DNAM-1 (CD226): DNAX accessory molecule−1
RNP: Ribonucleoprotein
TALENs: Transcription activator-like effector nucleases
GMP: Good manufacturing practice
NPM1c: Nucleophosmin 1 (mutated/cytoplasmic variant)
MM: Multiple myeloma
BCMA: B-cell maturation antigen
GPRC5D: G protein-coupled receptor, Class C, Group 5, Member D
HL-60: Human Leukemia cell line 60
sNKG2D-L: Soluble NKG2D ligand
CIML: Cytokine-induced memory-like
HDACi: Histone deacetylase inhibitor
GD2: Disialoganglioside 2
GPC3: Glypican-3
sPD-L1: Soluble PD-L1
c-MET: Mesenchymal–epithelial transition factor
EpCAM (CD326): Epithelial cell adhesion molecule
MUC-1: Mucin 1
HER2: Human epidermal growth factor receptor 2
CEA: Carcinoembryonic antigen
MCTS: Multicellular tumor spheroids
CD25: Cluster of differentiation 25
CRC: Colorectal cancer
MSLN: Mesothelin
STING: Stimulator of interferon genes
EGFR: Epidermal growth factor receptor
EGFRvIII: Epidermal growth factor receptor variant III
TNBC: Triple-negative breast cancer
HNSCC: Head and neck squamous cell carcinoma
PDAC: Pancreatic ductal adenocarcinoma
CCCR: Chimeric co-stimulatory conversion receptor
DLL3: Delta-like ligand 3
SCLC: Small cell lung cancer
LUAD: Lung adenocarcinoma
CD56: Cluster of differentiation 56
CD107a: Cluster of differentiation 107a (LAMP-1)
B7-H3: B7 Homolog 3
CD73: Cluster of Differentiation 73 (ecto-5'-nucleotidase)
GBM: Glioblastoma
CSPG4: Chondroitin sulfate proteoglycan 4
PB: Peripheral blood
CB: Umbilical cord blood
HPCs: Hematopoietic progenitor cells
HSCs: Hematopoietic stem cells
TDEs: Tumor-derived exosomes
PGE₂: Prostaglandin E2
TIM-3: T-cell immunoglobulin and mucin domain-containing protein 3
CTLA4: Cytotoxic T-lymphocyte-associated protein 4
A2: Adenosine A2 receptor
ROS: Reactive oxygen species
MAJESTIC: MRNA adeno-associated virus–sleeping beauty joint engineering of stable therapeutic immune cells
